# Functional Evaluation of Patients Undergoing Multiple Joint Replacements: A Retrospective Study of 50 Patients with a Minimum of Six Months of Follow-up

**DOI:** 10.7759/cureus.830

**Published:** 2016-10-15

**Authors:** Shubhranshu S Mohanty, Nandan Mishra, Prateek Patil, Ajinkya Desale

**Affiliations:** 1 Professor & Unit Head, Department of Orthopaedics, Seth GS Medical College & KEM Hospital, Mumbai, India; 2 Department of Orthopaedics, KEM Hospital, Mumbai; 3 Orthopedics, LTMMC and GH, Mumbai

**Keywords:** polyarthritis, multiple joint arthroplasties, sf-36 scores, physical component scores, mental component scores

## Abstract

Introduction: Polyarthritis is a challenging condition that an orthopedic surgeon faces in day-to-day practice. Some of the conditions where multiple joints are affected are rheumatoid arthritis, osteoarthritis, and ankylosing spondylitis. Multiple joint afflictions can cause severe impairment in the quality of life, which leads to a significant socioeconomic burden on the family and society. Joint replacement is considered as a treatment when severe joint pain or dysfunction is not alleviated by conservative management. Total joint arthroplasty remains one of the most commonly performed and universally accepted operative interventions for such patients.

Materials and methods: Fifty patients were invited into the study. All patients included in the study were 18 years of age and older and had undergone two or more joint replacements with a minimum of six months duration from the last surgery. The data was collected during the preoperative and postoperative periods through patient records and questionnaires. The Short Form 36 Health Survey Questionnaire (SF-36) scores were generated from an online application that is readily available on the official website SF-36 scoring system. The results were compared, analyzed, and tested for significance using the Wilcoxon signed rank test.

Results: The highest incidence of multiple joint replacements appears to be in the age-group of 51 - 70 years (52%), the mean age of patients being 51.7 +/- 14.4 years. The ratio of female to male patients was 1.6:1. On comparison of preoperative and postoperative (six months) physical component and mental component scores, the differences were found to be significant (p-value: < 0.01). This finding is irrespective of the diagnosis, gender, or age of the patient.

Conclusion: In the study conducted on 50 patients, we found out that multiple joint arthroplasties are fruitful surgeries. The procedures are efficient in reducing the disabilities seen in patients with polyarthritis of various causes and improving the overall quality of life. We strongly recommend multiple joint arthroplasties to patients with severe disability. However, adequate medical management plays an equally important role to improve the overall results. Well-designed and larger studies are required to establish the treatment protocols and order of surgeries in patients with differing causes of polyarthritis.

## Introduction

Polyarthritis is the systemic involvement of multiple joints of the body (five or more in number). The disability produced by polyarthritis varies from mild to severe, depending on the etiology, severity of individual joint involvement, the pattern of joint involvement (small joint/large joint), and the number of joints involved. The conditions where multiple joints are affected are rheumatoid arthritis [1-2], avascular necrosis (AVN) of the femoral heads [3], ankylosing spondylitis [4-5], osteoarthritis [6], psoriatic arthritis, and hemophilic arthropathy [7]. Multiple joint affection causes severe impairment in the quality of life, which leads to a significant socioeconomic burden on the family and society.

Polyarthritis is a challenging condition that an orthopedic surgeon faces in day-to-day practice. Joint replacement is considered as a treatment when severe joint pain or dysfunction is not alleviated by conservative management. Total joint arthroplasty remains one of the most commonly performed and universally accepted operative interventions for such patients. In this era of the nuclear family and independent survival, physical mobility is of paramount importance. In recent years, with an increasing life expectancy and advances in geriatric medicine, the scenario of joint replacement surgeries has changed significantly. An increasing number of younger patients [8] are undergoing joint replacement for pathologies like rheumatoid arthritis and ankylosing spondylitis.

This study becomes more important in the Indian scenario because of widespread ignorance and apathy among patients and a deprivation of a strong social support base, coupled with a poor public health awareness and infrastructure. Such patients present after multiple years of neglect with grossly deformed joints. With the advent of newer prostheses and a better understanding of the disease process, it is possible to provide a pain-free, mobile joint to the patient through multiple joint replacement surgeries. Since multiple joint replacements are not commonly performed procedures, few studies are available in the literature regarding such procedures [9], with respect to the technical difficulties that are frequently encountered and functional status in the long run. For the purpose of calculating the improvement in the quality of life, there are different scoring methods, such as the Harris hip score [10] and the Oxford hip and knee score [11], which are specific to a particular joint. This study attempts to investigate the short-term outcomes of multiple joint replacements in patients with polyarthritis by using the Short Form 36 Health Survey Questionnaire (SF-36) scores [12-13].

## Materials and methods

A series of 50 patients who underwent multiple joint arthroplasties (two or more joints replaced with prosthesis) were studied from January 2013 to November 2015. The aims of the study were to identify the demography of patients undergoing multiple joint arthroplasties and to assess the short-term functional results of the same.

The Ethics Committee of the Seth GSMC and KEM Hospital, Parel, Mumbai issued approval IEC (I)/OUT/2013/1248/15. A written informed consent was obtained from all patients before enrollment into the study.

All the patients included in the study were 18 years of age and older and had a minimum follow-up of six months duration from the last surgery. The patients who developed severe medical or other systemic complications in the postoperative period requiring ICU care or extended hospitalization were excluded from the study.

After enrollment, the patients were investigated radiologically with preoperative x-rays (both anteroposterior and lateral views) of the involved joints. The preoperative functional status was assessed using SF-36 questionnaire, and the SF-36 scores (physical component scores (PCS) and mental component scores (MCS)) were generated using an online database. The patients were also investigated hematologically with erythrocyte sedimentation rate, C-reactive protein, other routine blood investigations, and specific blood investigations to titrate the activity of the disease process. A rheumatologist's opinion was taken for the medical management. The patients in whom the activity of the disease process was under control with appropriate medical treatment, were posted for anesthesia fitness. Once the patients were declared fit for anesthesia, they were posted for elective sequential or staggered surgeries.

All patients were operated upon by a single surgeon under antibiotic coverage and reviewed independently. During the postoperative period, the intravenous antibiotics were continued for at least five days, followed by oral antibiotics. Adequate analgesia was provided, and the patients were put on an intensive postoperative physical therapy program.

Postoperatively, the patients were investigated radiologically with immediate postoperative x-rays. The patients were discharged after suture removal of the last surgical wound. The patients were regularly followed up during the postoperative period at one, three, and six months. The radiological and hematological investigations were repeated at each follow-up visit. A strict monitoring for the disease activity was carried out. At six months of follow-up, the functional status was reassessed using the SF-36 questionnaire with the online generation of SF-36 scores (physical component scores and mental component scores). The SF-36 health survey questionnaire was developed to create two scales that provide glimpses into the physical and mental functioning, as well as the overall health-related-quality of life. The results were assimilated, analyzed statistically, and tested for significance using the Wilcoxon signed rank test.

## Results

Fifty patients who had undergone two or more than two joint replacements, operated by a single surgeon of the institute, were regularly followed up. Postoperative patients were given the SF-36 questionnaire in their vernacular language and asked to mark the answers. The SF-scores were generated and compared to the preoperative SF-36 score. The following observations were made.

**Figure 1:**
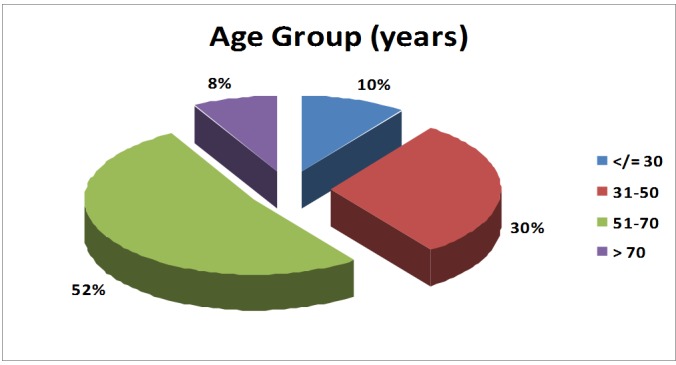
Pie chart showing the age-group distribution of patients undergoing multiple joint replacements. The pie chart shows the age-group distribution of the patients undergoing multiple joint arthroplasties. The highest number of the patients belonged to the age group of 51-70 years, followed by 31-50 years. Younger patients (less than 30 years of age) presenting for multiple joint replacements are seen less commonly.

The highest incidence of multiple joint replacements appeared to be in the age-group 51-70 years. The mean age of patients in the study was 51.7 +/- 14.4 years (Table 1) (Figure 1).

**Table 1:** Age group incidences of patients who underwent multiple joint replacements The table shows the number of patients in each age group undergoing multiple joint replacements. It can be seen from the table that the maximum number of patients (more than 50%) belonged to 51-70 years of age group, which is the most common age group with osteoarthritis. The next common age group is 31-50 years. Younger patients (less than 30 years of age) presenting with polyarthritis for multiple joint arthroplasties are seen less  commonly. The mean age of presentation of patients in our study group is 51.7 years.

Age group (yrs)	N	%
</ = 30	5	10.0%
31 - 50	15	30.0%
51 - 70	26	52.0%
> 70	4	8.0%
Total	50	100.0%
Mean age: 51.7 +/- 14.4 years (20 - 75 yrs)		

Of the 50 patients, 31 were females and 19 were males. The ratio between female and male was 1.6:1.

**Figure 2:**
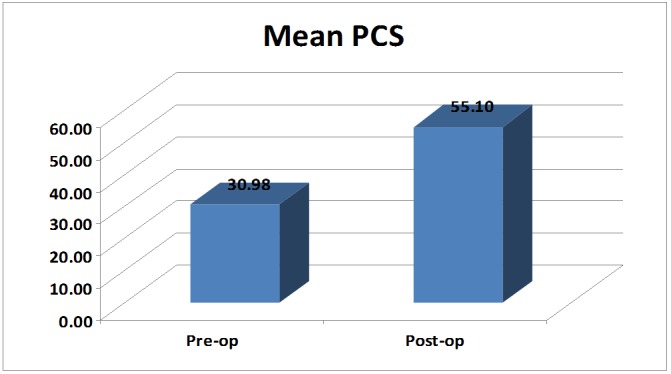
Bar graph showing mean preoperative and postoperative physical component scores (PCS). The figure shows the mean preoperative and postoperative PCS of 30.98 and 55.10, respectively. The changes were found to be significant.

The mean preoperative physical component score was 30.98 with a standard deviation (SD) of 3.52. The postoperative mean value was 55.10 with an SD of 5.41. The difference between the means of physical component scores was significant (p-value: < 0.01) (Table 2) (Figure 2).

**Table 2:** Mean Preoperative and Postoperative Physical Component Scores (PCS) of the Study Subjects The table shows the mean preoperative PCS of the study subjects, which is 30.98 with a standard deviation of 3.52. During the postoperative period, the study subjects demonstrated a mean PCS of 55.10 with a standard deviation (SD) of 5.41. The change in the mean physical component scores after surgery at six months of follow-up was significant with a p-value of less than 0.01.

PCS	N	Mean	SD	p-value
Pre-op	50	30.98	3.52	< 0.01
Post-op	50	55.10	5.41	

 

**Figure 3:**
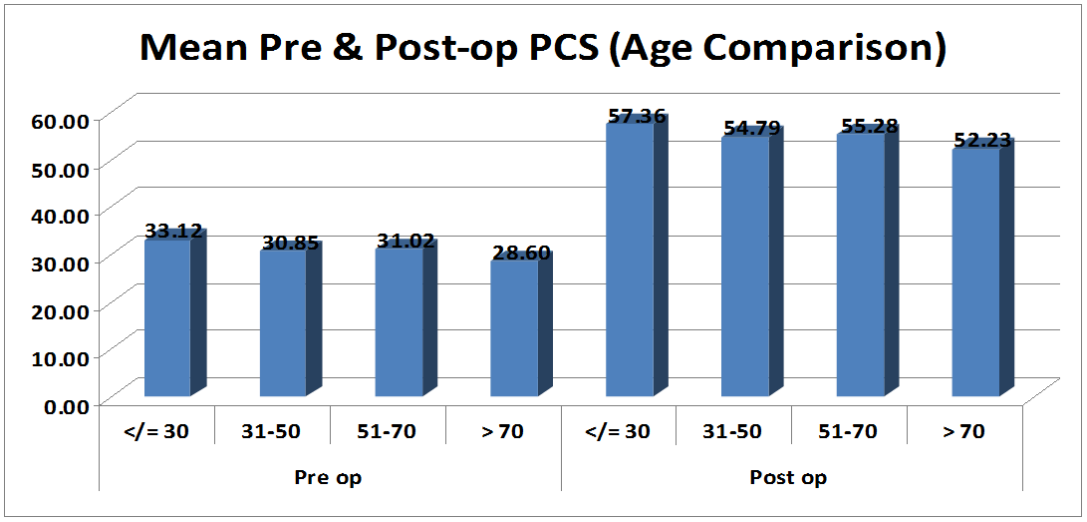
Bar graph showing preoperative and postoperative mean physical component scores (PCS) in the different age groups. The bar graph shows mean PCS during the preoperative and postoperative periods in each age group. The graph shows an improvement in the scores irrespective of age group, suggesting that age has little role in treatment decision-making in a patient with polyarthritis and significant disability.

The patients were divided into four groups according to their age (< 30 years, 31-50 years, 51-70 years, > 70 years). The differences in means of physical component scores were calculated for patients in each age group and the results were found to be significant (p-value: < 0.05) in each age group (Figure 3).

**Figure 4:**
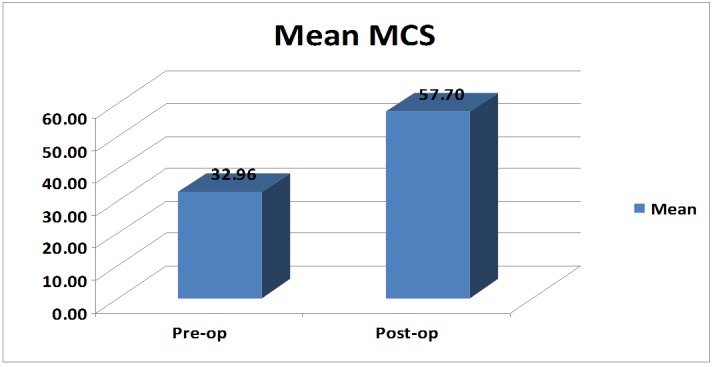
Bar graph showing mean preoperative and postoperative mental component scores (MCS). The figure shows the mean preoperative and postoperative MCS of 32.96 and 57.70, respectively. The changes were found to be significant.

The mean preoperative mental component score was 32.96 with an SD of 4.07, and the postoperative mean value was 57.70 with an SD of 5.05. The difference between the means of mental component scores was significant (p-value: < 0.01) (Table 3) (Figure 4).

**Table 3:** Mean Mental Component Scores (MCS) in the Study Subjects The table shows the mean preoperative MCS of the study subjects, which is 32.96 with a standard deviation (SD) of 4.07. During the postoperative period, the study subjects demonstrated a mean MCS of 57.70 with an SD of 5.05. The change in the mean MCS after surgery at six months of follow-up was significant with a p-value of less than 0.01.

MCS	N	Mean	SD	p-value
Pre-op	50	32.96	4.07	< 0.01
Post-op	50	57.70	5.05	

 

**Figure 5:**
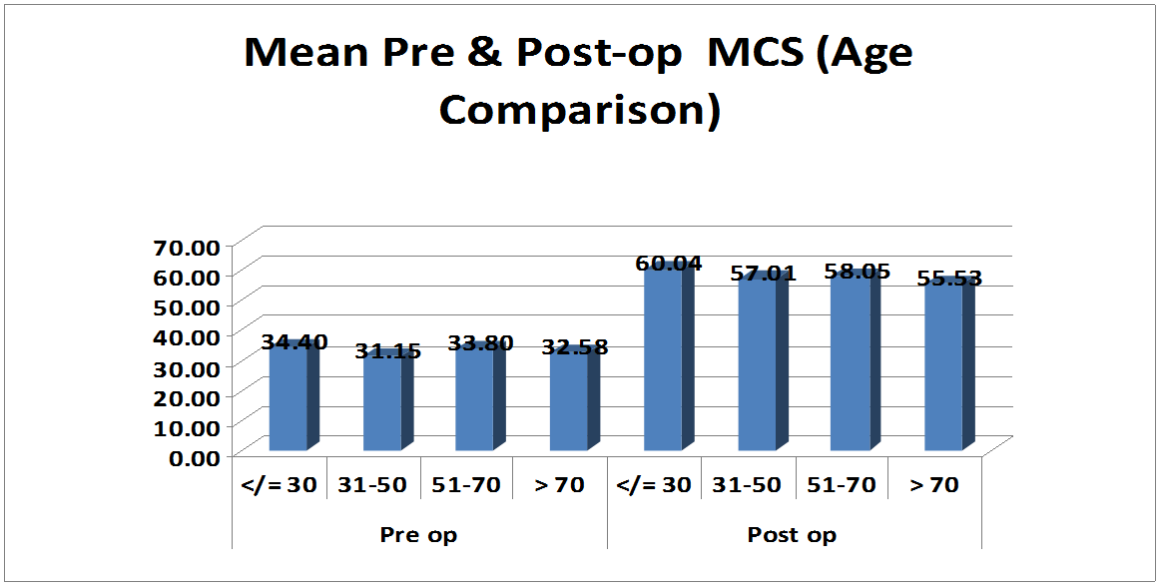
Bar graph showing preoperative and postoperative mean mental component scores (MCS) in the different age-groups. The bar graph shows mean MCS during the preoperative and postoperative periods in each age group. The graph shows an improvement in the scores irrespective of age group, suggesting that age has little role in treatment decision making in a patient with polyarthritis and significant disability.

The patients were divided into four groups according to their age (< 30 years, 31-50 years, 51-70 years, > 70 years). The differences in means of mental component scores were calculated for patients in each age group and the results were found to be significant (p-value: < 0.05) in each age group (Figure 5).

**Figure 6:**
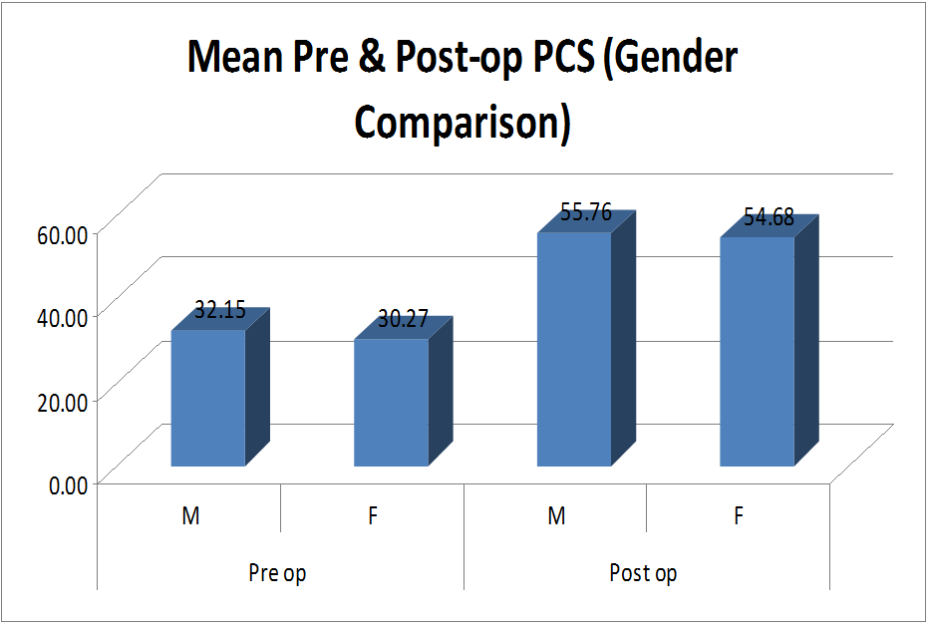
Bar graph showing mean preoperative and postoperative physical component scores (PCS) in males and females. The bar graph shows significant improvement of PCS during the postoperative period irrespective of the sex of the patient, suggesting that sex of the patient does not affect the functional outcomes of multiple joint arthroplasties in patients with polyarthritis and significant disability.

**Figure 7:**
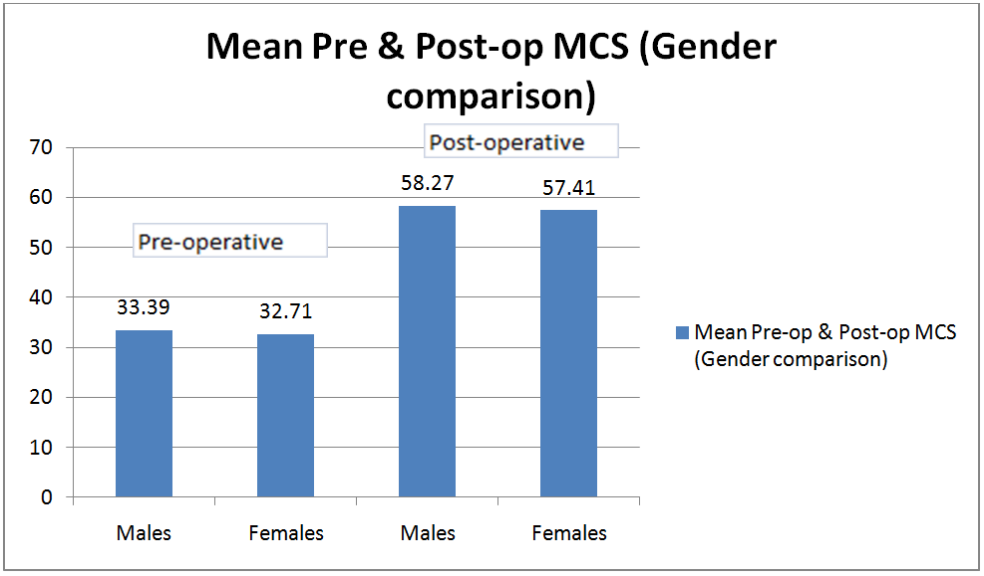
Bar graph showing mean preoperative and postoperative mental component scores (MCS) in males and females. The bar graph shows significant improvement of MCS during the postoperative period irrespective of the sex of the patient, suggesting that sex of the patient does not affect the functional outcomes of multiple joint arthroplasties in patients with polyarthritis and significant disability.

When the patients were divided into two groups according to their gender, the differences in means of preoperative and postoperative scores were significant in both males and females. This was true for both the physical component scores (Figure 6) and the mental component scores (Figure 7).

## Discussion

The overall incidence of multiple joint replacements is progressively increasing possibly because of advanced medical care, increasing demands, and affordability of the patients. Providing a functional joint that improves the lifestyle of the patient is a challenging task. Multiple joint replacements have yielded satisfactory results. A planned rehabilitation program has been equally important. The end functional result is still dependent on the diagnosis, the severity of the disease process, the number and pattern of joint involvement, the preoperative status of mobility, and the duration of the disease process.

All the patients in the study were 18 years and older (18 to 80 years). The mean age of the subjects in the study was 51.7 years, and the majority of the patients were in the group of 51 to 70 years. Similar studies have been carried out in the past in patients with a specific pathology [14-16]. Therefore, the age group in these studies was largely defined. In our study, we have included multiple pathologies for which the patient is undergoing surgery, which explains the large range of age distribution. The mean age of patients undergoing bilateral hip replacements for AVN hip was 46.4 years in accordance with the literature regarding age distribution of AVN [3]. In the studies where the outcome of total hip replacement was assessed in patients with ankylosed hip joints (ankylosing spondylitis) [4-5], the mean age was 37 years. In comparison, our study group of patients with ankylosing spondylitis had a mean age of 27 years. The mean age in our entire study is on the higher side as the majority of patients in the study had age-related osteoarthritis of the weight-bearing joints.

In this study, 31 patients out of the 50 were females and 19 patients were males. The female to male ratio was 1.6:1. Since the study included a large majority of cases with osteoarthritis, we observed a greater number of female patients in the study sample. Moreover, rheumatoid arthritis is common in females, and our study included eight females out of nine cases of rheumatoid arthritis who underwent multiple joint replacements.

It was observed that the mean physical component score improved from 30.98 - 55.10 and the mean mental component score improved from 32.97 - 57.28. The results show that there was a significant improvement in the lifestyle of the patients who have undergone multiple joint replacements as assessed by the SF-36 scores. This postoperative improvement was possible despite difficult circumstances related to anesthesia, positioning, and surgical approach of these grotesque deformities and the problems of physical rehabilitation faced by these patients.

A few studies have been carried out in the past on multiple joint replacements. The studies related to multiple joint arthroplasties are a six-year follow-up of multiple joint replacements in rheumatoid arthritis patients in 1994 [14], long-term follow-up of prosthetic joint replacement in hemophilia in 1986 [16], and the impact of total joint replacement in 2003 [15]. All of the studies have shown that the lifestyle has improved significantly in the postoperative period and the patients have become less dependent on the family and caregivers. However, the studies mentioned above have not considered the mental component score, which is possible only with the SF-36 scoring system.

The differences in the means of physical component scores were calculated for each age group, and the results were found significant (p-value: < .05) in all age-groups. We observed the highest improvement in the postoperative scores in younger patients (age group less than 30 years). The mean physical component scores improved from 33.12 - 57.36 and the mental component from 34.4 - 60.04. This was possibly due to the most active phase of the life being less than 30 years of age. With a good postoperative rehabilitation, these patients recover faster and tend to go back to normality earlier than their older counterparts. However, patients over the age of 70 years also showed a significant improvement in their SF-36 scores (mean PCS from 28.6 - 51.23 and mean MCS from 32.58 - 55.53). The above results suggest that the age of a patient should not be a strict criterion for decision-making in the management of these patients. Other factors, like preoperative mobility, comorbidities, and the status of the soft tissues around the joint, must be considered in the decision-making process. There was no significant difference between the improvement in males and females. Both the groups showed nearly equal improvements in both PCS and MCS.

The limitations of this study were a small sample size, a short-term follow-up, and a diverse group of patients with differing pathologies. Therefore, it is difficult to extrapolate the study results to the larger population. Larger, well-designed studies are required to establish the treatment protocols and order of surgeries in patients with differing causes of polyarthritis. Long-term outcome assessment studies with longer follow-up periods are required to determine whether results achieved in the study are sustainable.

## Conclusions

After the study conducted on 50 patients with polyarthritis, we have concluded that multiple joint arthroplasties are reliable and effective treatment modalities for patients with polyarthritis and severe disability. The SF-36 scores are useful tools for assessment of the disability in patients with polyarthritis and also for assessment of outcomes after surgery. The improvements were seen irrespective of the diagnosis, age, or gender. However, the best results are achieved only with a dedicated team approach that includes surgeons, rheumatologists, anesthetists, physiotherapists, hospital staff, and family support. The medical management of the disease process is also an important factor, especially in the younger patients who have a progressive systemic inflammatory polyarthritis rather than osteoarthritis, which is mainly seen in the older patients. Without adequate medical management, supervised physical therapy, and rehabilitation, the results would be compromised and might not improve the overall quality of life of the patients. The observations revealed that there is no age limitation to dictate the management. If there is an indication for the patient to undergo the procedures and the patient is fit to undergo such procedures, we strongly recommend multiple joint arthroplasties for a better quality of life, especially in those with severe disabilities.
